# How Purposeful Adaptive Responses to Adverse Conditions Facilitate Successful Auditory Functioning: A Conceptual Model

**DOI:** 10.1177/23312165251317010

**Published:** 2025-03-16

**Authors:** Timothy Beechey, Graham Naylor

**Affiliations:** 1 Hearing Sciences–Scottish Section, Mental Health and Clinical Neurosciences, School of Medicine, 170718 University of Nottingham, Glasgow, UK

**Keywords:** hearing, communication, listening, adaptive behavior, noise

## Abstract

This paper describes a conceptual model of adaptive responses to adverse auditory conditions with the aim of providing a basis for better understanding the demands of, and opportunities for, successful real-life auditory functioning. We review examples of behaviors that facilitate auditory functioning in adverse conditions. Next, we outline the concept of purpose-driven behavior and describe how changing behavior can ensure stable performance in a changing environment. We describe how tasks and environments (both physical and social) dictate which behaviors are possible and effective facilitators of auditory functioning, and how hearing disability may be understood in terms of capacity to adapt to the environment. A conceptual model of adaptive cognitive, physical, and linguistic responses within a moderating negative feedback system is presented along with implications for the interpretation of auditory experiments which seek to predict functioning outside the laboratory or clinic. We argue that taking account of how people can improve their own performance by adapting their behavior and modifying their environment may contribute to more robust and generalizable experimental findings.

## Introduction

Humans are highly adaptable, able to survive and thrive in a wide range of environments. When faced with adverse conditions in the external environment, or bodily impairments, a person can change the environment and their own behavior to facilitate successful functioning. Humans are rarely limited to a single course of action but will select different adaptive strategies depending on the nature of the task, the environment in which it must be performed, and the individual's resources, skills, experience, and preferences. Measures of high-level auditory functioning, such as listening or communication, which seek to predict performance or disability outside the clinic or laboratory usually quantify how functional outcomes are affected by changes in the environment. But, we argue, to more accurately predict such functioning, there is a need for an increased focus on how successful auditory functioning is achieved and maintained. Considering what people do to achieve and maintain auditory functioning—including trade-offs between different adaptive strategies and the choices people make when faced with adverse communication conditions—is a prerequisite for making accurate predictions of the effects of hearing impairment and noise outside laboratory and clinical settings.

Perceivers function in sensory and social environments (Gibson, 1966, 2015; Valenti & Gold, 1991). As the environment changes, perceivers are affected by these changes, typically as a function of impairment and other individual factors. However, understanding the characteristics of a person and their environment is not sufficient to fully understand hearing disability. Changes in the environment trigger responses from perceivers; importantly, the effects of these responses on perception can alter functional outcomes. A person may adapt their behavior to the environment. A person also may interact with and alter their environment, adapting it to their own needs (Heft, 2007; Kirsh, 1996).

In a real-world listening scenario such as the often referred to cocktail party (e.g., Pichora-Fuller et al., 2017), a person's level of speech intelligibility is impacted by their hearing and cognition; by the physical environment including the level and type of background noise, room acoustics, and characteristics of the speech signal being listened to; and by other psychosocial factors including personality and perceived social norms. However, a person at a cocktail party is not a passive receiver of signals; they may attempt to improve their perception in various ways. A listener may adapt to the environment, by (for example) turning their head (Brimijoin et al., 2012; Grange & Culling, 2016), blocking an ear which is dominated by noise, moving closer to the talker they are trying to hear (Miles et al., 2023), or directing increased attention to visual information (Hadley et al., 2019; Yi et al., 2013). When such behaviors are allowed during an experiment, and adopted by participants, they can improve observed performance. When available behaviors are not employed, performance may suffer. For example, reporting results of a spatial release from a masking study in which participants could freely move their heads, Grange and Culling (2016, p. 709) state “some listeners did not move … Consequently, they lost track of the speech earlier than other listeners.” A listener may also adapt the environment to their own needs, such as by asking the talker to speak louder, to repeat or rephrase an utterance, or to move with the listener away from a noise source. On this view, hearing disability is a product of four broad factors: impairment, environment, psychosocial factors, and the things a person does which affect their own perception.

Central to this approach to auditory functioning is the concept of *purposeful behavior* (see Table 1 for a summary of terminology). Responses to changes in the environment such as those described above are purposeful to the extent that they are directed by goal attainment: achieving or maintaining a particular state (Rosenblueth et al., 1943). Purpose tends to be relatively fixed across time. For example, a person may have the purpose of communicating with another person; while the environment may change throughout the course of a conversation, purpose may remain fixed: in this case, communication. While people do revise their purposes, such as when achieving the current purpose is too hard, purpose is likely less variable than might be presumed on the basis of variation in observable behaviors (see examples provided by Arocha, 2021). In many circumstances, goal attainment is also remarkably stable. For example, despite changes in the environment which impinge on perception, people are often able to continue to communicate (Beechey et al., 2020). As environmental conditions change, behavior changes in compensation, resulting in relatively fixed performance.

**Table 1.** Summary of Terms.

Term	Definition
Active behavior	Behavior which is energized from within the behaving organism by stored energy, not by an external force.
Adaptive response	A type of purposeful behavior directed by achievement or maintenance of successful functioning in a task environment.
Affordances	Opportunities for behavior made available by the environment.
Ecological capacity	The extent to which an organism can adapt to a given environment sufficiently to perform a given function.
Negative feedback	A causal loop in which the output of a system becomes input to the system such that increased output leads to decreased output and decreased output leads to increased output.
Purposeful behavior	A type of active behavior directed by goal attainment and moderated by negative feedback.
Task environment	A subset of the larger environment in which a specific task is performed.
Vicarious functioning	When multiple functions may be combined or exchanged because they each act in the same direction in relation to attainment of a goal.

The aim of this paper is to describe an ecological^1^ model of adaptive responses to adverse auditory conditions which has at its heart purposeful behavior, and which unifies a range of seemingly disparate areas of hearing science into a single dynamic system. This model may be useful for researchers in the design and interpretation of experiments which seek to model auditory functioning outside the laboratory. Adaptive responses to adverse auditory conditions encompass cognitive, physical, and linguistic domains. Within the cognitive domain, listening effort (understood here to subsume effort both in “de-noising” a percept and in exploiting context to improve guesses) is perhaps the most familiar type of response to adverse auditory conditions, and much progress has been made toward better understanding cognitive responses to adverse auditory conditions. Physical and linguistic behaviors share two properties with cognitive listening effort. Firstly, like cognitive listening effort, physical and linguistic behaviors constitute costs; they require expenditure of energy stored in the body, and carry potential social costs which may arise from behaviors such as standing too close or speaking too loud. Secondly, like cognitive listening effort, physical and linguistic behaviors serve a purpose; the maintenance of effective auditory functioning. Because cognitive, physical, and linguistic responses to adverse conditions serve the same purpose, they form part of an adaptive *system* in which they may be combined with, and substituted for, each other. As a consequence, we argue here that each category of adaptive response should be treated with equal importance in the definition and measurement of auditory functioning and disability. This does not imply that each of these response modalities has equal effectiveness in every situation. Considering cognitive, physical, and linguistic responses to adverse auditory conditions as facets of a single adaptive system may facilitate a better understanding of auditory function, dysfunction, and disability.

Our model of adaptive responses shares with existing literature on cognitive listening effort a focus on *capacity*. Since each of the three response types involves expenditure of energy, each is necessarily dependent on finite resources. But our approach to capacity is more general: we view capacity in ecological terms as an individual's overall capability to adapt to the demands of the physical and social environment in performing particular auditory tasks. This ecological approach to capacity provides a novel view of hearing disability as an insufficient capacity to adapt to a given sensory environment to enable successful auditory function. Similarly, a person with normal hearing thresholds who despite all efforts to adapt, is unable to hear well enough to function successfully in adverse acoustic conditions, also has insufficient capacity to adapt to their environment.

We first provide a brief typology of responses to adverse conditions in the cognitive, physical, and linguistic domains, relating this to the framework of the World Health Organization (WHO) International Classification of Functioning and Disability (ICF) (World Health Organization, 2001). This is followed by an explanation of purposeful behavior and its role in achieving stable functional outcomes in a changing environment. Next, we outline how diverse responses to adverse conditions can share a common purpose—known as *vicarious functioning* (Brunswik, 1952)—and describe how different responses are selected by *affordances* (Gibson, 1966); the opportunities for action made available by the environment. This is followed by an outline of ecological capacity and how this relates to functioning and disability. We then present a generalized framework of adaptive responses related to auditory functioning and its consequences for the definition and measurement of hearing disability. We discuss this model of adaptive responses in the context of related approaches in hearing science and other fields. Finally, we present some implications for the design and interpretation of experiments.

## Adaptation and Auditory Functioning: A Brief Typology

In this section, we give a nonexhaustive outline of the range of things people do as part of, or in support of, auditory functioning. This section describes the range of phenomena we believe must be accounted for in a model of adaptive auditory functioning.

Auditory functioning may be classified into four hierarchical categories based on levels of functioning more generally specified within the WHO ICF (World Health Organization, 2001), including *hearing*, *listening*, *comprehending*, and *communicating* (Kiessling et al., 2003). *Hearing* is defined as “essentially a passive function” (Kiessling et al., 2003, p. S93) in terms of attention and intention, though *hearing* is active in the sense of neural activity. Each higher-level auditory function starting with *listening* is considered to require active engagement such as attention and intention. *Listening* and *comprehending* are defined as one-way processes involving reception of auditory signals, with *comprehension* distinguished from *listening* in terms of reception of meaning. *Communicating* is the only category of auditory functioning defined within the ICF framework as involving a two-way process: the bidirectional transfer of information in which two (or more) people receive and transmit information to each other.

The definitions of auditory functions put forth by Kiessling et al. (2003) are closely linked to cognitive functions, particularly above the level of *hearing*. Such cognitive operations fall broadly into two categories: (i) cognitive resource allocation and use in the case of *listening*; and (ii) information processing in the case of *comprehending* and *communicating*. Linguistic processes are implicit in the definition of *comprehending* and *communicating* as these functions frequently relate to the understanding and production of human language. However, consideration of linguistic processes in Kiessling et al.'s definitions is limited to information processing, such as “matching a sound input to appropriate stored knowledge” (p. S96) in the case of *comprehending*, and “optimizing the exchange of information” (p. S96) in the case of *communicating*.

This strong focus on *within the head* auditory and cognitive processes fails to capture many aspects of auditory-related functioning. In particular, the definitions provided by Kiessling et al. (2003) overlook many of the ways that people function *within the physical and social environment* when they are free to do so. Considering how people hear, listen, comprehend, and communicate within their environment reveals a broader range of processes.

When *listening* in everyday situations, a person frequently has the opportunity to *do* things to improve their own signal reception. For example, Borschke et al. (2024) report that hearing aid users frequently use behavioral strategies including turning down background noise such as music, moving closer to a conversation partner, or asking a conversation partner to speak louder, more clearly, or to repeat an utterance. The necessary utility of such responses is illustrated by the fact that they typically are not employed when they are not useful or effective. A familiar example of this utility of responses to adverse conditions has been observed during experimental conditions in the form of reversals of changes in physiological responses in the most challenging conditions where the listening task may be impossible and the listener disengages (e.g., Richter et al., 2008; Zekveld et al., 2018). The variety of things a person may do to improve their signal reception is far broader than the responses reflected in such physiological examples. Consider a person in their own home listening to music on the radio: while a person in this scenario might respond to difficulty receiving the signal by using cognitive strategies such as increased allocation of attention, they may respond in multiple other ways. The listener may move closer to the radio, thereby improving the SNR, or may turn up the volume of the radio with the same effect. The listener may also close a window to reduce noise from outside or silence some other noise source within the house, similarly improving the SNR. The listener may achieve the same effect by picking up the radio and moving with it away from sources of noise. This illustrates how, in addition to cognitive operations, the listener can take *physical* actions to change their own position within the environment relative to sources of signal and noise and also physically change the environment. This example also points to the need for broader conceptions of active and interactive functioning. A person may actively respond to auditory difficulty in ways that go beyond cognitive functioning, such as by taking physical actions. Similarly, adaptive responses may be interactive to the extent that they alter any aspect of the environment that leads to altered perception, not only through verbal interaction with another person. A related Bayesian approach has been proposed by Vasil et al. (2020) in which it is argued that people adapt their behavior in order to find evidence confirming an adaptive prior belief that their mental states are aligned with those of other people. By adapting in this way, Vasil et al. (2020) argue that people adapt to their environment and simultaneously adapt their environment to their own needs.

In a *comprehension* scenario, a similar array of strategies can be employed. If a person were listening to a news report rather than to music, each of the strategies described above would be relevant, in addition to cognitive operations related to attention and information processing enumerated by Kiessling et al. (2003). In a different comprehension scenario such as a person attempting to understand speech while watching television, still other strategies could be usefully employed. For example, to access redundant information a person may direct their gaze to nonauditory cues such as visual speech cues or subtitles. Or, to improve the SNR they may adjust the television volume, make use of an assistive listening device, or even cup an ear, improving the SNR in the mid-frequencies (Barr-Hamilton, 1983).

*Communication* affords the greatest variety of behaviors, reflecting the hierarchical nature of functioning described by the ICF. In addition to cognitive operations and physical actions which may directly affect SNRs, linguistic behaviors can be used to affect the signal. Through verbal interaction, a person may, for example, cause a conversation partner to produce Lombard speech or to repeat or rephrase an utterance (Beechey et al., 2018, 2020) or alter the timing of speech to take advantage of temporal gaps in noise (Aubanel & Cooke, 2013). A person engaged in interactive communication may also make use of an array of behaviors which signal affect including facial expressions, gestures, and production of prosodic cues such as changes in stress, intonation, and timing (e.g., Beechey, 2022; Corley et al., 2007) each of which may cause a conversation partner to adapt their own behavior in ways which aid communication.

Even at the level of *hearing* which, as defined by Kiessling et al. (2003), does not involve active engagement, a person may take deliberate actions to improve sensory performance in the future, a form of *temporal adaptation* (Kielhofner, 1977). For example, based on past experience of hearing difficulty, a person may choose to sit in a location where they will hear the doorbell, or move a device, or increase the volume setting of a device, to ensure that the device will be heard should it produce an alert or alarm at a future time, even when no specific auditory signal is expected, or listened for. Consider the example of a person increasing the volume of an alarm which will wake them from sleep the next morning. Such a course of action constitutes *planning*. Planning to hear differs crucially from listening in that it does not require attention to present sound. But, like listening, hearing can be affected by conscious, intentional, and physical actions directed by a purpose. Evidence for such prospective adaptation is mixed: Pasta et al. (2022) reported that some hearing aid users proactively select a program setting in anticipation of acoustic conditions. In contrast, in an ecological momentary assessment study of hearing aid wearers’ adaptations to environmental conditions. Borschke et al. (2024) found that anticipatory adaptations were reported rarely by participants.

A particular strength of viewing auditory functioning through the lens of the ICF framework is that it makes clear that even normal, unimpaired auditory functioning requires perceivers to *do* things. We can’t define normal or unimpaired auditory functioning as not needing to do anything: we all need to do things to function in the environment because the environment can exceed every organisms’ capacity at times (Boorse, 2009). For example, even a person with normal hearing thresholds may not be able to understand soft speech from another room. However, there is a need to expand consideration of the things people do as part of successful auditory functioning if we are to understand normal and disordered auditory functioning and make better predictions of the effects of adverse auditory conditions.

## Purposeful Behavior

Purposeful behavior is a type of *active* behavior directed by the minimization of the discrepancy between a current state experienced by a perceiver and a goal state, by potentially diverse means (Marken, 2021). Guidance or direction for such behavior necessarily comes from within the behaving organism. Consider again the person moving toward a talker: where the person moves to is based on achieving a goal state. If the goal state is a sufficient level of speech comprehension to understand a speech signal, the person's movement will be guided by their own level of comprehension. In practice, people often hold multiple purposes which may be hierarchical (one purpose serves another higher purpose) or in conflict. People must then sequence or prioritize behaviors which satisfy different purposes. However, for simplicity, we will often talk about purposes as if they are singular. For an organism to guide its own behavior in this way requires (i) an internal criterion defining the goal state, (ii) sensory input upon which to determine the discrepancy between the internal criterion and the current state, and (iii) *negative feedback* loops to modulate behavior. Rosenblueth et al. (1943, p. 19) argue that “[a]ll purposeful behavior may be considered to require negative feedback. If a goal is to be attained, some signals from the goal are necessary at some time to direct the behavior.” The combination of moderating negative feedback loops and corresponding comparison functions to detect discrepancies between the current state and a goal state at each level of the system is a hallmark of control engineering in the context of machines (Bennett, 1993, 2008) and Perceptual Control Theory in the context of living organisms (Marken, 2021; Powers, 1973a, 1973b).

Continuing with the example of a person moving toward a talker: initial movement toward the talker is prompted by sensory experience of poor speech comprehension and by a prediction, likely based on experience, that moving closer will help to improve speech comprehension; as the person moves toward the talker they are guided by continuous signals informing them of their level of comprehension; as they move, comprehension continuously improves, decreasing the discrepancy between current and goal states. On encountering an obstacle, the person may navigate around the obstacle to achieve the subgoal of not bumping into anything, a subgoal which may temporarily require the person to move away from the target talker, reducing comprehension, before continuing to move toward the talker until the person achieves the goal state of a sufficient comprehension and stops moving closer.

Negative feedback need not occur only over very short timescales while a person is receiving a signal. As in the example of taking preemptive actions to improve hearing in the future such as by increasing the volume of an alarm, the adaptive response may be to past experiences of not having heard. If the alarm is still not loud enough to wake the person from sleep the following morning they may then further increase the volume the next evening. Such preemptive actions do not simply change the moment of social or physical cost. Rather, they may change the nature of the auditory task and result in overall lower cost (Hutchins, 1995).

That adaptive behavior is guided by negative feedback in relation to an internal criterion does not imply that adaptive processes must be conscious (Wiener, 2019). King (1978) argues that when an organism is focused on achieving a goal, adaptation may be subconscious and unrecognized; that attention to the goal rather than the adaptive process is critical.

Because purposeful behavior acts, via moderating negative feedback, toward attainment of goals which are relatively fixed, outcomes resulting from purposeful behavior tend toward stable states. For example, Beechey et al. (2020) observed a stable ability to continue a conversation among participants who adapted their communication behavior to compensate for changes in environmental conditions. More generally, Arocha (2021) provides examples that illustrate how adverse events such as falling when walking occur far less frequently than might be expected because people adapt to the environment and maintain equilibrium.

How a particular purpose is determined may stem from a large number of factors. In the most general terms, a purpose might be specified by the type of auditory task being undertaken by an individual. For example, in a comprehending scenario, such as trying to understand an announcement at an airport, the purpose may be to correctly receive information needed to find the right gate; in a communication scenario, the purpose could be to correctly judge the honesty of an interlocutor. Important influences on purpose include states, characteristics and preferences of individuals, and motivations related to specific situations. These factors might affect both what a person's purpose is in a particular situation, and how durable that purpose is likely to be. For example, if a person is unable to hear an announcement at an airport and is worried that they may miss their flight, they may ask a stranger what was said in the announcement despite any embarrassment, to avoid the cost and stress associated with missing a flight. In contrast, a person who wishes to follow a conversation in a social context may choose not to ask a talker to repeat their utterance because they are more concerned about appearing “old.” In this case, a person may modify their purpose—from understanding speech to not drawing attention to themself—rather than adapt their behavior to achieve their initial purpose. Hence, the fact that people can adapt their behavior to achieve a purpose in a changing environment does not exclude the possibility that people may choose to change their purpose instead.

## Selection of Responses to Adverse Communication Conditions

People often appear to behave in variable and unpredictable ways. However, variance in behavior in daily life should not be seen as random. People can and do behave in different ways, but the *outcomes* people achieve in daily life are remarkably low in variance. Arocha (2021) gives the example of people commuting to their place of work: of the people who leave home for work each morning, we do not observe some sizeable minority randomly arriving somewhere else by accident.

Likewise, responses to adverse communication conditions are variable in terms of observable behavior. For example, in an adverse communication setting, a person trying to hear speech clearly can move closer to a talker, ask the talker to repeat an utterance, or direct cognitive resources to attend to the speech signal and suppress noise. These and many other behaviors can be combined and interchanged. The choices a person makes will depend on individual preferences and a sense of the likely costs and benefits of different courses of action. Combining and choosing between these behaviors is possible because they all serve a common purpose: improving speech understanding. This is an example of *vicarious functioning* defined as “the flexibility and exchangeability of pathways relative to an end” by Brunswik (1952, p. 675).

Recognition of the role of variable behaviors in achieving stable outcomes can be traced back to the Functionalist school of psychology (e.g., Dewey, 1896) and has long been considered a hallmark of human functioning. For example, when considering what would make a robot like a human in its functioning, Boring (1946, p. 180) argued that “There is little that will make our robot seem more human than this ability to choose one means after another until the goal is reached.” More recently, Toro et al. (2020) argue that open-ended capacity to adapt to the environment is a hallmark of a healthy-bodied person.

The use of different means of responding to adverse communication conditions is not simply a function of individual choice. The ways a person can adapt is a function of the opportunities made available by the task and the physical and social environment. A person may consciously choose between multiple available courses of action by, for example, considering the likely costs (cognitive, physical, and social) and benefits, but it is the task and the environment that ultimately select viable strategies. For example, Pryce and Gooberman-Hill (2012) describe situations in a residential care home, where difficulty hearing in noise is not resolved because residents have poor mobility and do not have the ability to control noise sources. Within Perceptual Control Theory, Runkel (2003, p. 6) describes the opportunity for action by a person in terms of: (i) a relevant *object* or *event* that can be acted upon; (ii) awareness of a *means* of affecting the object or event; (iii) *capability* of using those means to take appropriate action; and (iv) *belief* or *expectation* that the action has a reasonable chance of affecting the environment in the desired way. Similarly, Gibson (1966) outlined a theory of *affordances* within Ecological Psychology describing perception of the environment in terms of opportunities for action.

**Figure 1.**
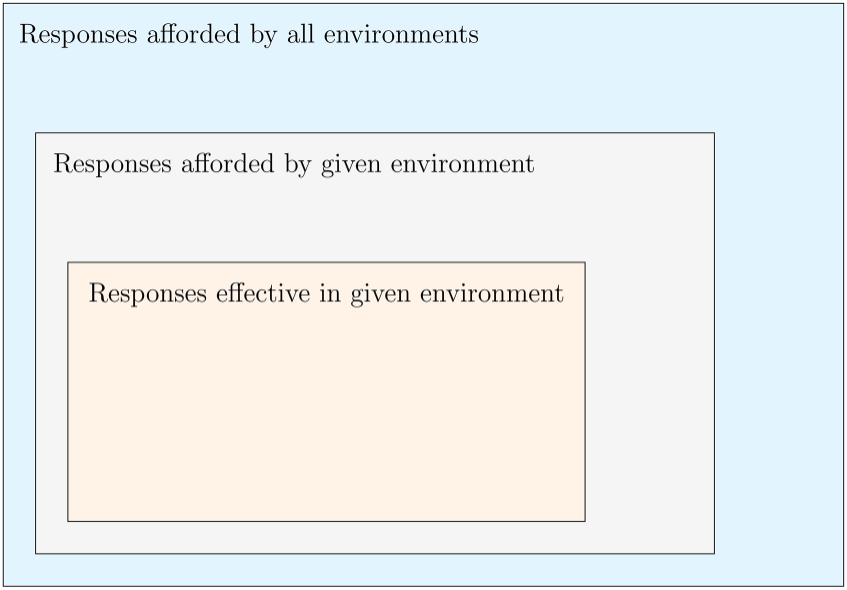
Relative availability and effectiveness of responses to adverse conditions.

It is not just the physical and social environment but also the task being performed in that environment that must be taken into account in order to understand how a person may respond to adverse communication conditions. The environment and the task can be combined into the concept of a *task environment* (Kirsh, 1996). We may, for example, distinguish between *comprehending* and *communicating* within the context of the ICF (Kiessling et al., 2003). In a comprehension scenario such as trying to understand an announcement at a train station, a person may move closer to a loudspeaker. A person may also direct attention to better comprehend speech. In contrast, in a communication scenario, a live, copresent talker makes verbal interaction a viable response to difficulty understanding speech. While a purpose can, in principle, be achieved through multiple means, in practice different means are available and effective in different task environments. This is represented schematically in Figure 1.

## Ecological Capacity

The environment exceeds every organism's capacity in certain ways (Boorse, 2009). Our functional range is greatly enhanced through the use of technology, but no matter how much we adapt ourselves to the environment or the environment to ourselves, there will be some challenges we cannot overcome. Importantly, the range of conditions in which an individual is able to function is a product not only of that individual's characteristics, including any hearing loss or other impairments; and current state, such as fatigue or anxiety; but also their adaptation to environmental conditions. Even individuals with normal hearing cannot rely on passively receiving signals. The apparent ease with which people can understand speech in ideal conditions belies the complex processes required to achieve this feat (Heald et al., 2016; Heald & Nusbaum, 2014; Norris & McQueen, 2008). That something like speech perception seems so easy is a result of the things we *do* rather than things not done. This holds equally for receptive speech perception and interactive communication.

On this basis, the difference between hearing disability and normal auditory functioning is not whether an individual needs to do things which may be regarded as effortful to function successfully. Rather, we argue that hearing disability is characterized by a need to do *more* in order to successfully adapt to the obstacles posed by the environment. Functional limitations result where the environment exceeds a person's capacity to adapt in either instantaneous or cumulative terms. Hearing impairment makes the task of adapting to the environment less surmountable. For example, high-frequency hearing impairment increases the SNR needed for speech recognition. As a result, a person with hearing impairment is likely to need to adapt more than a person with normal hearing to achieve equivalent functioning in a given environment. Hearing impairment does not decrease capacity to adapt, it increases the amount of adaptation required to meet environmental and task demands. In this way, hearing impairment makes it more likely that a given environment or task will exceed a person's capacity to adapt. It also increases the likelihood that adaptations which would be required to compensate for the environment would be perceived as not worthwhile or desirable. For example, a person with hearing impairment might need to ask for more repetitions or to move closer to a talker than is perceived to be socially acceptable. A person in this situation may feel that asking for repetitions makes them appear old, an outcome that they could consider to be worse than not understanding an utterance.

This conceptualization of capacity supplements the approach to capacity typically assumed in the cognitive listening effort literature (e.g., Pichora-Fuller et al., 2016). Where the cognitive listening effort literature is concerned with the allocation of finite cognitive resources (Kahneman, 1973; e.g., Knowles, 1963; Moray, 1967), ecological capacity is related to within-individual adaptive fit to the environment (e.g., Kirsh, 1996).

## A Model of Adaptive Responses to Adverse Auditory Conditions

**Figure 2.**
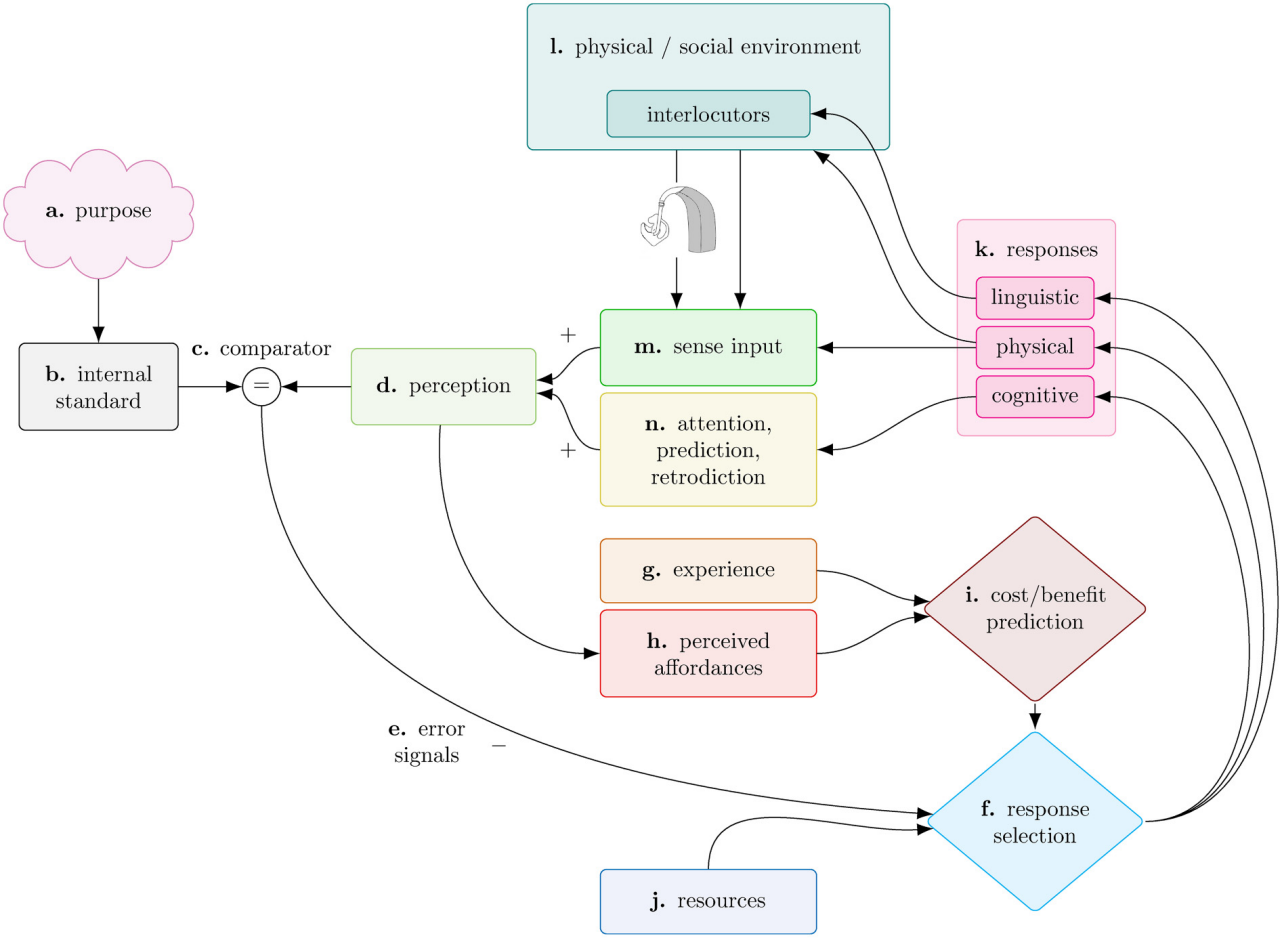
Model of adaptive responses to adverse conditions. Arrows indicate direction of flow through the system. Opposite signs indicate that the system acts as a negative feedback loop, minimizing discrepancies between the internal standard (b) and perception (d).

Adaptive responses to adverse auditory conditions can be described at a functional level by the dynamic negative feedback system depicted in Figure 2, which is directed by the purpose of achieving and maintaining auditory functioning. This model does not attempt to encompass every detail of reality. Rather, like many existing models, this model is a deliberate simplification aimed at illuminating general issues (Bowman & Targowski, 1987; Epstein, 2008; Hoel, 2017; Smaldino, 2017). It is this level of specificity that we believe will be a useful starting point for considering how common clinical and experimental methods relate to the dynamic, adaptive behaviors that constitute real-world hearing, listening, and communicating. Every element of the model we propose could be elaborated with far more detail and, indeed, this greater detail exists in the literature. It is the combination of elements and their connections in the model which forms the basis of the more general message that people may adapt to a changing environment in a range of ways rather than simply being affected by the environment. We do not, for example, aim to replicate low-level neural explanations of adaptation that can be found in the neuroscience literature (e.g., Friston, 2010; Friston et al., 2010, 2017), nor do we aim to account for all the complexities of interactive communication between people or the details of how individual characteristics may affect choices about adaptive behavior.

Stable performance is achieved via moderating negative feedback loops. Goal attainment is the reward for expenditure of energy and social capital, which constitute the cost (Fairclough & Houston, 2004; Hallberg & Carlsson, 1991). Adaptive responses to adverse auditory conditions are exhibited in diverse ways including any combination of: cognitive operations, such as allocation of cognitive resources; physical movement and interaction with objects in the environment; and eliciting behavior from people in the environment through linguistic expression and nonverbal communication. The selection of particular modalities of adaptive responses is a function of available resources, predicted costs and benefits, affordances and demands of the task environment, along with individual propensities.

In undertaking an auditory task in a given environment, a perceiver who has a *purpose* (Figure 2(a)) possesses an *internal standard* (Figure 2(b).) informed by that purpose against which *perceptual signals* (Figure 2(d)) are continuously compared. For example, in attempting to comprehend speech produced by a conversation partner across a table in a noisy restaurant, the perceiver may judge whether the current SNR is sufficiently positive to allow communication. The system works to ensure that over time characteristics of the perceptual signal match the internal standard, regardless of whether the auditory task is listening, comprehending, or communicating (see Vasil et al., 2020 for an alternative Bayesian approach specific to interactive communication). When *comparison* (Figure 2(c)) of the perceptual signals against the internal standard reveals a discrepancy, *error signals* (Figure 2(e)) are produced. Error signals trigger production of *responses* (Figure 2(k)). The form that responses take is not deterministic; responses are informed by experience (Figure 2(g)), which incorporates knowledge such as social rules and conventions, and *affordances* (Figure 2(h)), those actions perceived to be possible and considered likely to be effective given the task and the physical and social environment. Experience and affordances each inform the predicted cost and benefit of different behaviors (Figure 2(i)). The *resources* (Figure 2(j)) the perceiver possesses, and the predicted cost and benefit of different behaviors influence the forms responses take.

The responses produced by the perceiver can affect subsequent sensory input (Figure 2(m)) directly via changes to the physical and social environment (Figure 2(l)) and via modification of cognitive processes which affect perception, such as attention, and prediction or retrodiction of speech signals (Figure 2(n)). Cognitive responses cannot affect the environment but may improve signal reception (Price & Bidelman, 2021; Reuter-Lorenz & Cappell, 2008; Vaden et al., 2013; Wingfield & Grossman, 2006). Physical responses may affect signal reception, such as improving SNR as a result of moving closer to the signal source (Weisser et al., 2021), or may affect the environment with flow-on effects on signal reception, such as closing a window to reduce noise from outside. A hearing device, if present, will affect the signal from the environment (Figure 2(l)) received at the external ear (sense input, Figure 2(m)). Where hearing devices are optimally fitted, they should improve perception of auditory signals and reduce discrepancies between perception (Figure 2(d)) and the internal standard (Figure 2(b)). As a result, a person may not need to adapt to the environment as much as they would in the absence of the device. Linguistic responses only affect the signal indirectly via affecting the people in the environment, such as asking a talker for a repetition resulting in modifications of morphosyntactic structure, vocal level, or timing (Aubanel & Cooke, 2013). Where responses cause a property of interest, such as intelligibility of a signal, to become more similar to the internal standard, reducing the discrepancy and associated error signals, the impetus to produce further responses is reduced. For example: if the perceiver requests a repetition which results in understanding of the utterance, the perceiver will then cease to request further repetitions of the same utterance; if a perceiver moves closer to a talker to improve the SNR they will stop moving closer once a sufficiently favorable SNR is achieved. This expectation is consistent with empirical findings of behavior of pairs of people engaged in conversation in noise presented by Miles et al. (2023) demonstrating that people first respond to the environment through physical movement and changes in vocal level, then maintain relatively constant interpersonal distance and vocal effort levels. This moderating effect identifies the model as a *negative* feedback system.

Like cognitive models of effort (e.g., Kahneman, 1973; Pichora-Fuller et al., 2016), this model of adaptive responses includes a *resource* component, reflecting the necessarily finite resources any individual possesses to carry out cognitive, physical, or linguistic responses to adverse auditory conditions. Resources here are defined broadly to encompass: cognitive resources, such as working memory and attention; physical resources, such as bodily ability to perform particular movements, and metabolic energy to power such movements; and knowledge and skills such as native-level mastery of a language. However, resources are not equivalent to, nor the sole determinant of *capacity to adapt* to the environment. Whereas resources are a characteristic of individuals at a given time, capacity to adapt is a function of an individual in combination with their task environment. For example, a person who has the mobility to close a window and shut out noise may not be afforded that strategy if everyone appreciates the window being open because the room is too hot. In such a scenario a person may have insufficient capacity to overcome the demands of the environment, despite ample resources. On the other hand, if a person possesses insufficient resources to produce responses that are in principle afforded by the task environment, the result is also a limited capacity to adapt.

When responses are produced by the perceiver and the sensory input changes, availability of resources, opportunities for action, and the environment itself also change, informing further responses. Throughout the feedback process, what remains constant is the internal standard, informed by the perceiver's purpose. A different internal standard comes into effect only once the purpose has been revised. The fixed nature of the internal standard is represented in Figure 2 by its position outside any feedback loops. Where adaptive responses to the environment through cognitive, physical, or linguistic means do not sufficiently reduce the discrepancy between the perceiver's internal standard and the incoming signal, the result may be revision or abandonment of the purpose, that is, giving up (Wu et al., 2016; Zekveld & Kramer, 2014).

A realistic model of behavior must contain multiple hierarchical feedback systems. Each level of purpose corresponds to a distinct level of adaptation. The model illustrated in Figure 2 represents adaptation at the scale of an individual interfacing with the external environment in the fulfillment of familiar auditory functions such as hearing, listening or communication. The model is intended to generalize across types of auditory task. While a receptive listening task is functionally quite different from an interactive multiperson conversation, what is common in these disparate tasks is that individuals adapt their behavior to the environment, depending on current affordances. In the case of conversation, each participant can be expected to operate according to the model illustrated in Figure 2.

But this model is only part of the story, it does not cover everything people do, even within the context of auditory tasks. It is a view of adaptation at a particular scale. It depends on lower-level feedback systems, such as those controlling muscle contractions necessary for producing effortful speech or physical movement. This model must also be linked to the larger cognitive system of an individual perceiver, including long-term memory, and the larger context of dynamic interaction between individuals, such as during conversation, which demands additional cognitive functions beyond the scope of this model, including theory of mind and alignment with interlocutors.

## Related Theories and Approaches

Each of the component concepts that make up the model illustrated in Figure 2 is derived from existing theories. What is novel in the current approach is the particular synthesis of these existing ideas.

The primary influence on the approach outlined here is *Perceptual Control Theory* (Marken, 2021, 2002; Powers, 1973a, 1973b) which developed from control engineering (see Bennett, 1993 for a review) and Cybernetics (Rosenblueth et al., 1943; Wiener, 2019) with its focus on the use of negative feedback systems to maintain steady states in machines, and Functionalist psychological approaches (e.g., James, 1995) which emphasized organisms’ adaptation to their environment. Perceptual Control Theory models organisms as “living control systems” (Marken, 2021) analogous to physical control systems such as thermostats, but with the crucial innovation of placing the criterion which defines the goal state inside the organism, as in the internal standard (Figure 2(b)), rather than externally (Powers, 1978).

However, our approach differs from Perceptual Control Theory in a number of ways. Perceptual Control Theory defines *perception* in broad terms that do not closely align with the definition of perception within hearing science and other perceptual fields. Within Perceptual Control Theory perception is not limited to sensory signals but rather is closer to an awareness of a current state (Marken, 2021). Importantly, Perceptual Control Theory posits that a perceiver does not directly perceive the environment, instead perceiving the environment indirectly as disturbances on its own behavior. It also does not consider that an organism may adapt the environment, focusing instead on adaptation of an organism to environmental disturbances (Marken, 1990).

While we have drawn on ideas from control engineering due to the intuitive nature of negative feedback as a basis for understanding adaptation to changing environments, our model is, in many ways, analogous to a more recent theory known as active inference (Friston et al., 2017) which is based on the free-energy principle (Friston, 2010) and on the Bayesian Brain theory more generally (Knill & Pouget, 2004). Like Perceptual Control Theory and our own model, Active Inference and the free-energy principle are based on optimization. Where our model is driven by minimization of discrepancies between an internal standard and a sensory input, the Free Energy theory posits an optimization mechanism which minimizes prediction error by ensuring that input signals are sampled preferentially through adaptive behaviors so as to reduce discrepancies between sensory signals and expectations implied by a mental model. Through this optimization mechanism, the active inference approach also contends that organisms will produce different actions that serve to stabilize sensory performance in a changing environment.

Drawing on very different literature, Borg outlined in a series of papers (Borg, 2000, 2003; Borg et al., 1998, 2008) a model of communication with superficial similarities to Perceptual Control Theory and our own model of auditory adaptation. Borg's ecological model of communication represents communication as directly analogous to the biological system of interaction between an animal and a plant (Borg et al., 1998). Parallel to our use of negative feedback, Borg argued for the concept of “recycling” of resources and disposal of “waste” during the communication process. Similarly, Borg's model is characterized by a “preferendum,” defined as an ecological niche, which is analogous to the internal standard (Figure 2(b)) drawn from Perceptual Control Theory. While Borg's model shares with our own a loop and a criterion, our approach differs in its use of specifically *negative* feedback as a moderating force. More importantly, our model differs fundamentally from Borg's model in the inclusion of: (i) *purpose* as the fundamental driving force guiding adaptation; and (ii) *affordances* as the means by which the task environment selects for different forms of response.

## Implications

### Considering the Utility of Adaptation in Addition to its Cost

Responses to adverse auditory conditions have been studied and theorized within hearing science mainly in terms of cognitive listening effort (e.g., Pichora-Fuller et al., 2016; Rönnberg et al., 2013). While potential benefits of cognitive listening effort are acknowledged by some authors (e.g., Herrmann & Johnsrude, 2020; Matthen, 2016; Pichora-Fuller et al., 2016), there appears to be far greater emphasis on its cost. Listening effort tends to be conceptualized as a symptom of dysfunction rather than a tool to facilitate functioning. There is a focus on listening effort as something people experience rather than something people do. For example, Francis & Love (2020) provide a summary of definitions of listening effort, each of which equates listening effort with cost: either opportunity cost, since expenditure of effort consumes resources that could otherwise be used for other purposes; or as a physiological resource cost, depleting metabolic energy stores. However, regardless of the type of cost assumed, definition of cognitive listening effort only in terms of cost is conceptually problematic, since effort can also produce benefits. It is reasonable to consider the expenditure of more effort as necessarily worse than expending less effort only when increased effort has no further impact on functional performance. But greater expenditure of effort can have a positive effect on perception (e.g., Price & Bidelman, 2021; Reuter-Lorenz & Cappell, 2008; Vaden et al., 2013; Wingfield & Grossman, 2006). The other side of the cost equation is benefit. In this sense, there may be many scenarios in which more effort is to be preferred over less. It can be better to adapt to the environment than not to adapt if adaptation will positively affect functioning and an individual has the capacity to do so. Similarly, where we consider physical or linguistic responses that a person may need to produce to communicate in adverse conditions or in the face of hearing impairment, we should consider the benefits that flow from these behaviors rather than viewing them purely as burdens. For example, while it is clearly preferable to hear what a person says without needing to ask for a repetition, asking for a repetition may allow one to understand what was said.

### Design of Hearing Experiments

Hearing experiments are typically designed, like experiments in related fields, to control certain variables. In many cases such control has the purpose and function of reducing variance related to factors assumed not to be directly relevant to the question the experiment is designed to answer. Experimental control is also valuable for supporting causal inference by ruling out extraneous influences. For example, studies of speech perception in noise within hearing science typically seek to ensure that all test subjects are native speakers of the same language and even dialect. However, experimental control may also take the form of disallowing participant behaviors that might naturally be employed by a person in an analogous situation outside the laboratory. For example, in a listening experiment in the free-field participants are typically positioned at a fixed distance from a loudspeaker to control the level reaching the ears. But fixing the position of the perceiver also removes the possibility of a natural adaptive behavior: moving closer to the signal source to improve SNRs. Notable exceptions can be found in the spatial hearing literature where the effects of perceivers’ movement have been considered in many studies. For example, Wallach (1939) argued that head movement improves sound localization by helping to disambiguate spatial cues; Ashmead et al. (1995) reported that participants were able to more accurately perceive distance to a sound source when walking directly toward the source than when stationary, which made available cues to rate of change in sound pressure level; Speigle and Loomis (1993) showed that movement at an angle relative to a sound source provides motion parallax cues that enhance distance perception; and Grange et al., (2018) demonstrated that spatial release from masking can be increased by a head turn so that an ear is oriented toward a talker.

Experimental control to avoid extraneous variance and experimental control which constrains adaptive behavior is not generally considered separately in experimental design though they have different statistical effects: one reduces variance while the other increases bias (see Yarkoni & Westfall, 2017 for a discussion of the bias-variance trade-off). Consideration should be given during the design of hearing experiments to whether experimental control will act to reduce random variation or will systematically affect results in a way which biases them away from what might occur in the absence of control. If the object of study is auditory functioning corresponding to one of the functional levels defined by the WHO ICF, such as listening or communicating, where a perceiver's behavior can affect the signal they receive, removing this feedback process through experimental control is an instance of increasing bias. This may indeed reduce variance, but variance about a biased estimate. It should be noted, however, that while allowing adaptive behaviors may reduce bias in relation to performance (e.g., an intelligibility score or an SNR at which a target score is achieved) it introduces an additional need to measure the cost of any adaptive behaviors.

One dimension along which to consider the realism of a hearing experiment is the extent to which the experiment models the system under study like an inanimate physical system or an animate purpose-driven system. Where the former approach is taken, the result is a potentially misleading model of causation. In a controlled experiment, changes in the environment appear to cause changes in a measured quantity of interest, such as speech intelligibility. But in a feedback system causation is circular (Marken & Mansell, 2013). The result may be erroneous predictions. For example, Wallach (1940) argued that sound localization on the vertical plane had not been demonstrated by previous laboratory studies of auditory localization which employed fixed head positions because it is dependent on head movement.

### Distinguishing the Experimenter's Purpose from the Participant's Purpose

Hearing experiments seek to model perceptual and behavioral processes that, in reality, are characterized by the purposes of the people we test in our experiments (see Figure 2). However, experiments are often designed, and their results interpreted, according to the purposes of the experimenter. It is important to consider how these purposes may be at odds.

In fundamental terms, an experimenter often seeks sensitivity and specificity. For example, using an adaptive speech test (e.g., Killion et al., 2004; Nilsson et al., 1994; Plomp, 1986; Plomp & Mimpen, 1979) an experimenter may aim to determine a speech reception threshold (SRT). For the experimenter it is important that incorrect responses are distinguished from correct responses, and that both occur, to determine a threshold. Logically a threshold cannot be determined if all trials yield the same response category (correct or incorrect). If a test were to deliver such results it would be incapable of revealing differences between people or conditions. Such a test would provide no information to the experimenter (Bateson, 2002).

In contrast, the purpose of a participant undergoing speech intelligibility testing is likely to be to understand as much as possible, in line with the experimenter's likely instructions to them. Given this purpose, if the participant or patient were afforded the opportunity to adapt the environment—improving the SNR by increasing the speech level or decreasing the noise level—they would do so according to their purpose, with the result that their score may be higher. Participants are likely to make use of any behavioral responses which they (rightly or wrongly) perceive to be afforded by the experimental protocol, with the result that the experimenter may draw erroneous conclusions about auditory function. For example, the exertion of cognitive effort in response to hard conditions means that the experiment cannot be understood as exclusively concerned with perception.

One way to understand the mismatch between the purpose of the experimenter and the purpose of the person being tested is in terms of the location of the criterion used for determining discrepancies between current and goal states. In an adaptive speech-in-noise test, the criterion is set externally by the experimenter. While the SNR is affected by the responses of the participant, the endpoint of these adjustments is the percentage correct score specified by the experimenter.

## Conclusion

Humans are not passive receivers of sensory information, as definitions of auditory functioning within the WHO ICF framework (e.g., Kiessling et al., 2003) indeed make clear. Humans interact with their environment in the course of auditory functioning, changing the environment to suit their needs, and changing their own behavior to compensate for the environment. A realistic model of auditory functioning in the environment should account for the possibility of such adaptations. Unlike the study of low-level auditory mechanisms, measurement at functional levels such as environmental awareness, listening, speech comprehension, and communication may be unrepresentative if the purposes of perceivers and opportunities for perceivers to pursue those purposes are not taken into account.

We have outlined a model of adaptation to adverse auditory conditions which is centered around the concept of purpose. Through moderating negative feedback, an individual may employ variable behaviors to ensure fixed outcomes. This approach suggests a greater emphasis on the measurement of behavior associated with auditory functioning than has typically been considered within hearing experiments. While much productive work has been done to better understand cognitive responses to adverse auditory conditions, working toward concomitant levels of understanding of physical and linguistic adaptations may further advance our understanding of auditory function and disability.

## Notes

1. We use the term *ecological* in reference to functioning in the environment. This usage is distinct from use of this term to describe similarity of test conditions to a given environment.
